# Reconsidering Control Logic and Translational Interpretation in TNEA‐Based Modulation of Hippocampal Oscillations

**DOI:** 10.1002/advs.78149

**Published:** 2026-10-04

**Authors:** Changzhen Gong, Arthur Yin Fan

**Affiliations:** ^1^ American TCM Association Roseville Minnesota USA; ^2^ McLean Center for Complementary and Alternative Medicine Vienna Virginia USA

Re: TNEA Regulates Hippocampal Oscillation by Improving Inhibitory Synaptic Plasticity to Ameliorate Cognitive Impairment in Alzheimer's Disease

Guo et al. report that three‐needle electroacupuncture (TNEA) ameliorates cognitive deficits in 5xFAD mice by restoring theta–gamma oscillations via parvalbumin‐positive (PV^+^) interneurons and inhibitory synaptic plasticity [1]. The study is technically ambitious, data‐rich and its conclusion is very encouraging; however, we believe several conceptual and methodological issues warrant careful reconsideration before translational conclusions are drawn.

First, the definition of “non‐acupoint” control relies on millimeter‐scale spatial separation from the real acupoint on the mouse scalp. Given the small cranial dimensions, shared innervation, and electrical field diffusion in scalp tissues, a 1 mm displacement cannot reasonably be assumed to isolate distinct neural substrates. Electrical and mechanical stimulation at this scale is expected to converge onto overlapping peripheral and central pathways, challenging the biological plausibility of the control logic [2].

Second, the protocol involves repeated scalp needle insertion (20 sessions over four weeks). In mice, such repetitive penetration may represent a nontrivial degree of mechanical injury and subsequent repair, potentially engaging nociceptive, inflammatory, and neuroimmune signaling pathways known to influence hippocampal activity. These effects may not be entirely separable from the neuromodulatory mechanisms attributed specifically to TNEA, and they may not be fully controlled for in the current experimental design [3]. With this in mind, we respectfully suggest that future studies, if feasible, consider adding additional group(s) to evaluate the effects of needling itself across multiple mechanisms.

Third, there appears to be a degree of internal mechanistic inconsistency. Chemogenetic inhibition of PV^+^ interneurons abolishes the TNEA‐induced improvements in cognition and oscillatory activity, yet reductions in amyloid deposition and microglial activation are still observed. This apparent uncoupling may suggest that neuropathological improvement alone is insufficient to restore behavioral function, or alternatively, that PV^+^ interneurons serve a permissive rather than primary mechanistic role. Accordingly, the causal hierarchy among amyloid pathology, inflammation, interneuron function, and cognition appears to remain unresolved at this stage [1].

Fourth, alterations in theta–gamma power, coherence, and phase–amplitude coupling are repeatedly interpreted as mechanistic drivers of cognitive improvement. However, hippocampal oscillations are emergent network phenomena sensitive to arousal, novelty, movement, and stress. Without interventions that selectively manipulate oscillatory dynamics independent of acupuncture, causal directionality cannot be firmly established [4].

Finally, the translational implications warrant particular caution. The study implicitly suggests that therapeutic efficacy depends on millimeter‐level precision in scalp stimulation: electroacupuncture at the designated acupoints is reported to be effective, whereas repeated manual acupuncture at the same acupoints is ineffective. This distinction is especially problematic given that the needle dimensions used are relatively large for mice when compared with human clinical scale. Such conclusions contrast sharply with established clinical practice, in which scalp acupuncture targets functional regions rather than mathematical points. Extrapolating rodent scalp microgeometry to human clinical protocols therefore risks misinforming translational neuroscience and integrative medicine alike [5].

In summary, while Guo et al. provide valuable electrophysiological observations, uncertainties regarding control validity, mechanistic coherence, and biological plausibility limit the strength of the conclusions. Addressing these issues would substantially enhance the rigor and translational relevance of the work.

## Conflicts of Interest

The authors declare no conflicts of interest.
